# Dr. Gordon Smith

**Published:** 1833-10

**Authors:** 


Died, on the 15th of September, Dr. Gordon Smith, aged forty-one, the author of a Treatise on Medical Jurisprudence. He was a man of considerable talent, but somewhat eccentric in his habits, and, like many men of genius, was deficient in the art of prospering. It is painful to think that the last days of Dr. Smith were spent within the walls of a prison!

